# Unique molecular mechanisms for maintenance and alteration of genetic information in the budding yeast *Saccharomyces cerevisiae*

**DOI:** 10.1186/s41021-017-0088-6

**Published:** 2017-12-01

**Authors:** Sayoko Ito-Harashima, Takashi Yagi

**Affiliations:** Department of Biological Sciences, Graduate School of Science, Osaka Prefecture University, 1-2 Gakuen-cho, Naka-ku, Sakai, Osaka, 599-8570 Japan

**Keywords:** *Saccharomyces cerevisiae*, DNA damage response, dNTP pool, DNA repair, Non-homologous end joining (NHEJ), Homologous recombination (HR), Mutation, Nonsense suppressors, Phenotypic variation, Environmental adaptation

## Abstract

The high-fidelity transmission of genetic information is crucial for the survival of organisms, the cells of which have the ability to protect DNA against endogenous and environmental agents, including reactive oxygen species (ROS), ionizing radiation, and various chemical compounds. The basis of protection mechanisms has been evolutionarily conserved from yeast to humans; however, each organism often has a specialized mode of regulation that uses different sets of machineries, particularly in lower eukaryotes. The divergence of molecular mechanisms among related organisms has provided insights into the evolution of cellular machineries to a higher architecture. Uncommon characteristics of machineries may also contribute to the development of new applications such as drugs with novel mechanisms of action. In contrast to the cellular properties for maintaining genetic information, living organisms, particularly microbes, inevitably undergo genetic alterations in order to adapt to environmental conditions. The maintenance and alteration of genetic information may be inextricably linked to each other. In this review, we describe recent findings on the unconventional molecular mechanisms of DNA damage response and DNA double-strand break (DSB) repair in the budding yeast *Saccharomyces cerevisiae*. We also introduce our previous research on genetic and phenotypic instabilities observed in a clonal population of clinically-derived *S. cerevisiae*. The molecular mechanisms of this case were associated with mutations to generate tyrosine-inserting tRNA-Tyr ochre suppressors and the position effects of mutation frequencies among eight tRNA-Tyr loci dispersed in the genome. Phenotypic variations among different strain backgrounds have also been observed by another type of nonsense suppressor, the aberrant form of the translation termination factor. Nonsense suppressors are considered to be responsible for the genome-wide translational readthrough of termination codons, including natural nonsense codons. The nonsense suppressor-mediated acquisition of phenotypic variations may be advantageous for adaptation to environmental conditions and survival during evolution.

## Background

Yeasts are important microorganisms in human life because they have been used for fermentation to produce beverages and foods, such as beer, wine, sake, and breads. The budding yeast *Saccharomyces cerevisiae*, the genome project of which was completed in 1996 [1], has also been playing significant roles as a model organism in biological and medical sciences. *S. cerevisiae* is easy to culture, and many experimental procedures such as gene transfer techniques and biochemical and classical genetic analyses have already been established. The most attractive feature is that the DNA fragments introduced into *S. cerevisiae* cells are homologously recombined at the region with homology (plasmids or chromosomes) at an extremely high frequency. This characteristic enables genetic manipulations to be performed very easily such as the knockout of chromosomal genes. Yeast is a unicellular organism with a similar cellular structure to higher eukaryotes including humans. The functions and expression patterns of genes are highly conserved from yeast to humans, and many human genes were cloned by the complementation of yeast mutants. For instance, basic regulatory mechanism of cell cycle, which is associated with tumor formation in mammals, has been well established in *S. cerevisiae* [2, 3]*.* Although these characteristics firmly established *S. cerevisiae* as a model organism, a number of studies have shown the uncommon features of phenomena and molecular mechanisms that are specific to this yeast. The fission yeast *Schizosaccharomyces pombe* may be a suitable model for understanding the biological nature of higher eukaryotes, particularly humans. *S. pombe* is evolutionarily and functionally highly divergent from *S. cerevisiae.* A comparative genome study suggested that these two yeasts diverged approximately 300–400 million years ago [4]. In contrast to *S. pombe*, *S. cerevisiae* has simpler centromere structures, and quite different composition of factors for histone methylation and heterochromatin organization. Furthermore, *S. cerevisiae* lacks RNA interference (RNAi) pathway, and scarcely contains introns in its genes [1, 5–7]. However, divergent molecular mechanisms among organisms reveal how cellular machineries acquired a higher architecture during evolution. Furthermore, the unique characteristics of machineries may be applicable to applied biology such as drug discovery with novel mechanisms of action. In contrast to the mechanisms to maintain the fidelity of genetic information, living organisms accept genetic alterations, which may occur via multiple mechanisms, to survive against environmental stress. The maintenance and alteration of genetic information are associated with the capacity of DNA repair, and are inextricably linked to each other.

In the early part of this article, we review the unique molecular mechanisms of DNA damage response and DNA DSB repair specific to *S. cerevisiae*. We then discuss our previous study on genetic and phenotypic instability in a clonal population of clinically-derived *S. cerevisiae*. We elucidated the molecular mechanisms of this case, which are associated with the formation of tRNA-Tyr ochre suppressors and the strong position effects of mutation frequencies in eight tRNA-Tyr loci dispersed in the genome. The bias of mutation frequencies appeared to be due to differences in the efficiency of mismatch DNA repair and translesion DNA synthesis at different loci. Furthermore, ochre suppressors caused variable phenotypes among cells depending on the mutated locus. Another type of nonsense suppressor, *PSI* ^+^, a prion form of the translation termination factor in *S. cerevisiae*, also confers phenotypic variations in different strain backgrounds upon environmental stress. Nonsense suppressors that cause the genome-wide translational readthrough of termination codons and phenotypic variations may be advantageous for adapting to environmental conditions and be a driving force for evolution.

### Ribonucleotide reductase (RNR) and its regulator: Excellent biomarkers for DNA damage responses

**Fig. 1:**
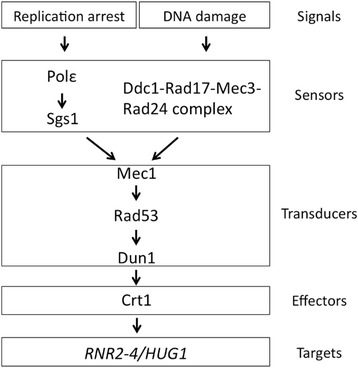
Checkpoint cascade in *S. cerevisiae*. DNA replication arrest and DNA lesions act as signals and are recognized by sensors to activate the checkpoint pathway. Sensors activate Mec1p kinase, followed by the consecutive phosphorylation of transducers and effectors to induce the expression of DNA damage response genes

**Fig. 2:**
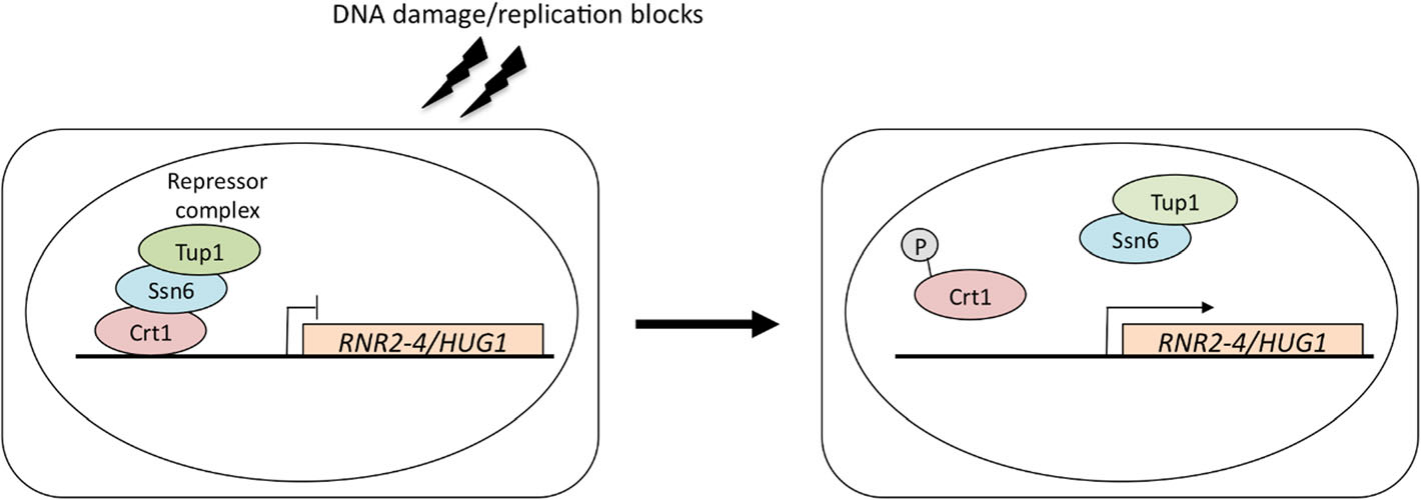
Regulatory mechanism of expression of DNA damage response genes by the checkpoint pathway. Upon the phosphorylation of Dun1p by the checkpoint pathway, Crt1p in the repressor complex bound to the promoter region of damage-inducible genes is phosphorylated. This leads to the dissociation of the repressor complex from the promoter to induce the expression of target genes

Upon DNA damage and DNA replication inhibition, eukaryotic cells activate cell cycle checkpoint functions. DNA damage checkpoint pathways are highly conserved between *S. cerevisiae* and humans [8]. The central components in checkpoint functions are ATM/ATR in humans and their homologs, Tel1p/Mec1p in *S. cerevisiae*, members of the phosphatidylinositol-3-kinase family [9, 10]. Signals received from the sensors for DNA damage and replication arrest are transduced through Tel1p/Mec1p, leading to the phosphorylation of the downstream transducers Rad53p and Dun1p protein kinases [8]. The activation of the checkpoint kinase cascade causes cell cycle arrest and the transcriptional induction of genes encoding subunits of RNR (*RNR2*, *RNR3*, and *RNR4*) and its regulator Hug1p (Hydroxyurea, Ultraviolet, and Gamma radiation 1) as well as DNA repair genes (Fig. 1) [11]. Figure 2 shows the mechanism of the transcriptional induction of DNA damage response genes mediated by Crt1p, one of the effector proteins that take part in the DNA damage and replication stress checkpoint pathway in *S. cerevisiae* [12]. Crt1p is a DNA-binding protein that binds to the promoter region of damage-inducible genes, including *RNR2*–*4* and *HUG1*, and represses their transcription by recruiting the general repressor complex Tup1p-Ssn6p [11]. In response to DNA damage or replication blocking, Crt1p is phosphorylated via the Mec1p-Rad53p-Dun1p kinase cascade and is then released from DNA, leading to the dissociation of the repressor complex and activation of target genes [11, 13]. The expression of *RNR2–4* and *HUG1* genes is induced by treatments with methyl methanesulfonate (MMS), hydroxyurea (HU), ultraviolet (UV), and γ rays [14–17]. In a previous study to examine responses to high-linear energy transfer (LET) ionizing radiation (IR), global gene expression in *S. cerevisiae* cells irradiated by three types of high-LET IR (fast neutrons, heavy ions, and thermal neutrons) and γ rays was investigated using a DNA microarray analysis. Five genes were induced by all forms of high-LET IR and γ rays, all of which are involved in the checkpoint pathway for DNA damage including *RNR2*, *RNR4*, and *HUG1*. The expression levels of these genes were up-regulated by 2.5- to 9-fold [18].

Deoxyribonucleoside triphosphate (dNTP) levels affected by RNR activity were increased more in *S. cerevisiae* during DNA damage responses than in *S. pombe* and mammalian cells [19–22]. Thus, changes in the expression levels of *RNR* genes and the *HUG1* gene appear to be marked. The induction of these genes upon DNA damage was also shown to be independent of the cell cycle stage, suggesting their properties as excellent biomarkers to detect DNA damage and replication-blocking agents in *S. cerevisiae*.

### Control of intracellular dNTP pools in *S. cerevisiae*: Tight regulatory mechanisms by three small proteins

RNRs are essential enzymes that catalyze the reduction of ribonucleotides to deoxyribonucleotides, the rate-limiting step in dNTP production, and play an essential role in de novo DNA synthesis [22]. The levels of dNTP pools are primarily regulated by RNR. It is suggested that the increased dNTP pools are required for optimal function of DNA polymerases to repair damaged DNA. Interestingly, the large pool expansions also increased mutation frequency [19].

**Fig. 3:**
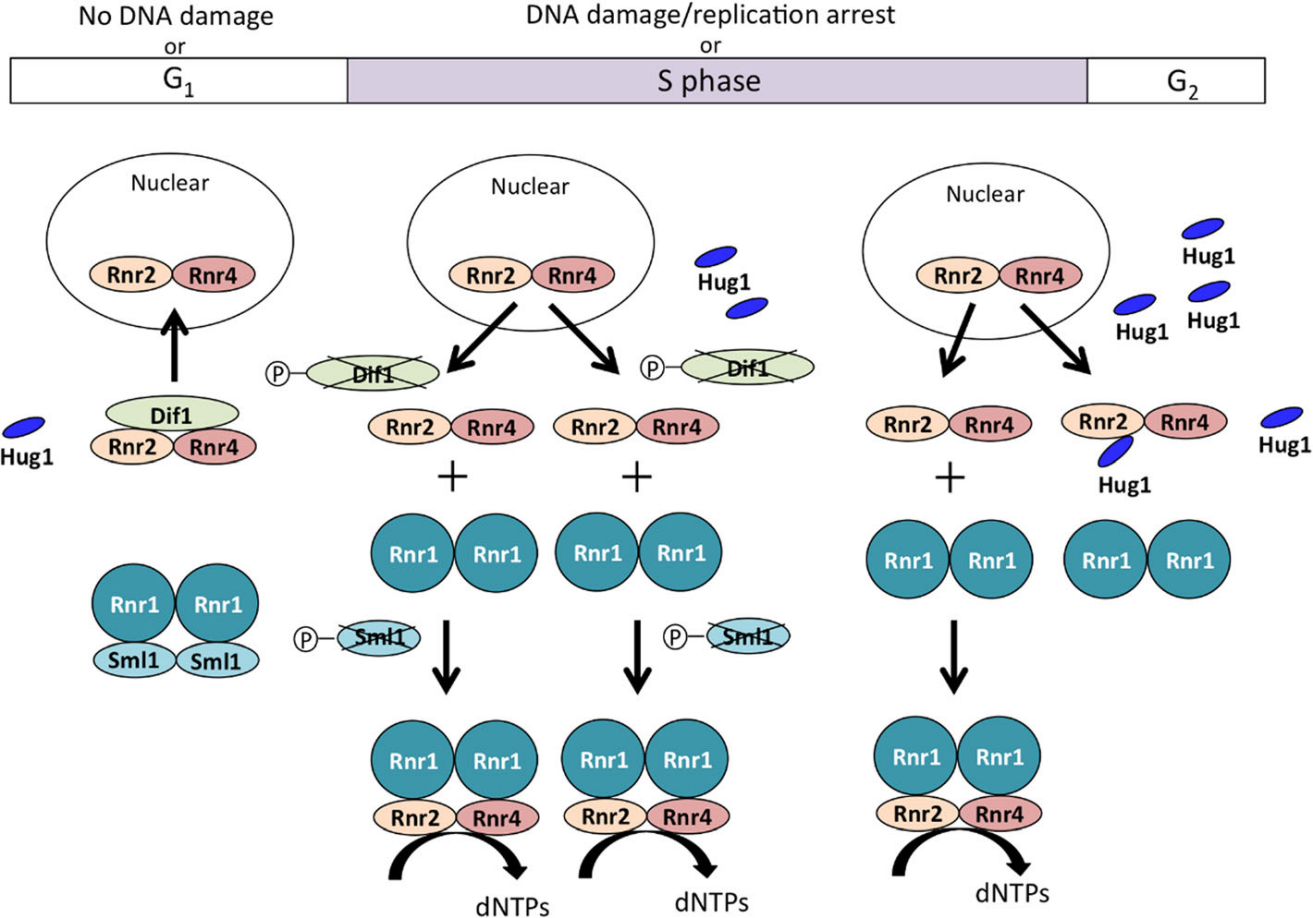
Regulation of RNR activity by three negative regulators. Sml1p binds to R1 (Rnr1p homodimer) in order to inhibit the catalytic activity of the R1 subunit, whereas Dif1p controls the nuclear localization of the R2 subunit (Rnr2p-Rnr4p heterodimer). Hug1p is induced in response to a replication block and DNA damage, and transiently attenuates increased RNR activity by inhibiting the association of R1 and R2 in the cytoplasm (see text)

In most eukaryotes, class Ia RNRs consist of a large R1 subunit (α_2_ homodimer) and small R2 subunit (β_2_ homodimer). In *S. cerevisiae*, four genes, *RNR1–4*, encode Rnr proteins: the Rnr1p homodimer forms R1, while the Rnr2p and Rnr4p heterodimer forms R2, which is an exceptional case in eukaryotes (Fig. 3) [23–25]. Rnr3p, a minor isoform of Rnr1p, is induced in response to DNA damage [26]. R1 is constitutively localized in the cytoplasm, whereas R2 is predominantly localized in the nucleus in the non-S-phase of the cell cycle. In response to S-phase entry or the activation of the DNA damage checkpoint, R2 is relocalized to the cytoplasm, where the RNR holoenzyme complex is formed to catalyze dNTP synthesis [27]. The enzymatic activity of RNR may be modulated by the binding of inhibitor proteins and dynamic changes in the subcellular localization of the R2 subunit.

**Fig. 4:**
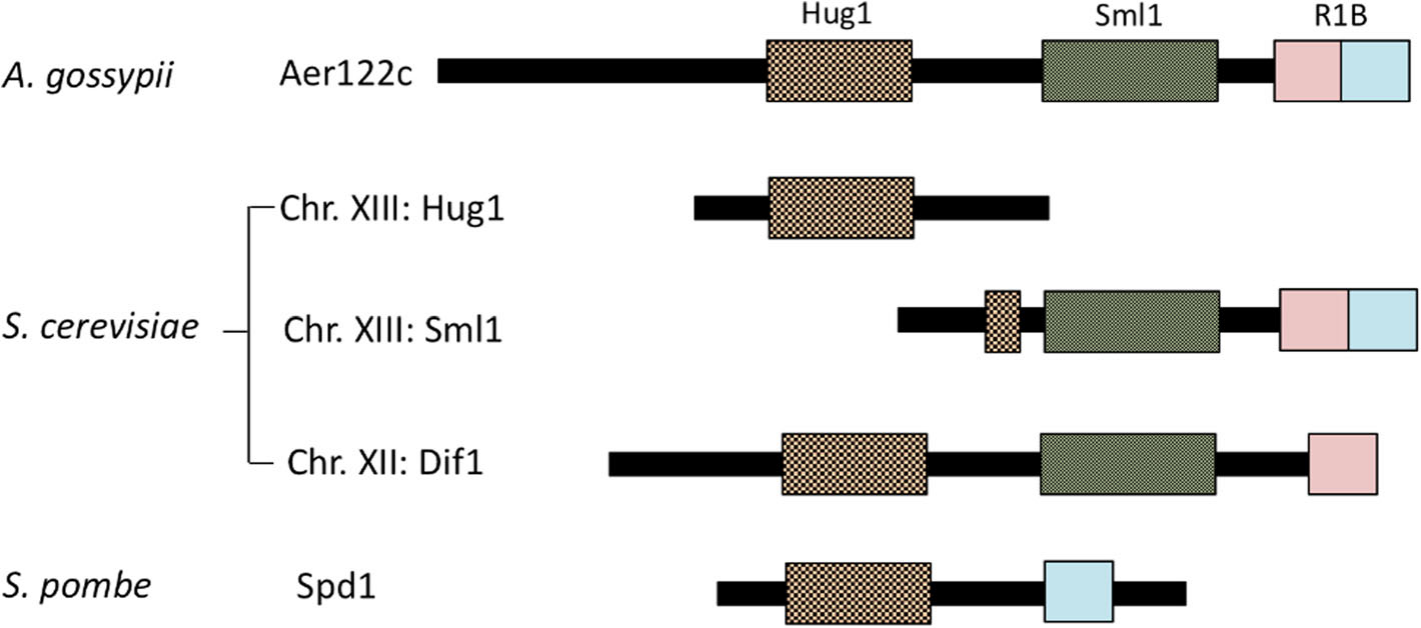
Structure of RNR regulators. *A* *gossypii* Aer122c is considered to be a prototype ancestral protein containing three domains: Hug1 (putative Rnr2p-Rnr4p binding domain), Sml1 (a degron for Smlp degradation after phosphorylation by Dun1p), and R1B (Rnr1p-binding domain consisting of two subdomains, which is inactive in Dif1p). Chromosomal localization of three genes encoding RNR regulators in *S. cerevisiae* is also depicted in the figure. *HUG1* and *SML1* genes, located in tandem on chromosome XIII, are transcribed separately

Three small intrinsically disordered proteins lacking fixed or ordered three-dimentional structure: Dif1p, Sml1p, and Hug1p, which are regulated by the checkpoint pathway, are involved in RNR regulation in *S. cerevisiae*. A synteny analysis suggested that these genes are derived from the same ancestral locus, diverging when *S. cerevisiae* underwent whole genome duplication during its evolutionary process [28]. The *DIF1* region, a putative ancestral gene on chromosome XII was duplicated and its paralog was split into the two separately transcribed genes, *HUG1* and *SML1*, located in tandem on Chromosome XIII (Fig. 4) [29]. These three protein factors are involved in the down-regulation of RNR activity, whereas the molecular mechanisms diverged. Dif1p binds directly to the R2 complex to drive nuclear import [29, 30]. Sml1p directly binds to cytosolic R1 and inhibits its catalytic activity [31–33]. During the S-phase and DNA damage response, Dun1p phosphorylates Dif1p and Sml1p, triggering ubiquitination-mediated degradation [34, 35]. The degradation of Dif1p and Sml1p allows the cytoplasmic localization of R2 and release of R1, respectively, followed by the association of R1 and R2 complexes in the cytoplasm to stimulate dNTP synthesis (Fig. 3). In *S. pombe*, Spd1 is important for the regulation of RNR. Spd1 binds to R1 and R2, affecting the localization of R2 and inhibition of the catalytic activity of R1 [36]. Spd1 appears to have the ability to combine Dif1p and Sml1p of *S. cerevisiae*.

Among the three regulatory proteins of *S. cerevisiae*, the expression of Hug1p only is induced in response to DNA damage and a replication block. Although the role of Hug1p in the checkpoint pathway and DNA damage-dependent dNTP synthesis has been suggested, the underlying mechanism remains unknown [13, 37]. Recent studies revealed that Hug1p negatively regulates dNTP synthesis by binding to Rnr2p [38, 39]. The induction of Hug1p in DNA damage responses was slower than Rnr3p, and Hug1p was found to be enriched in the cytoplasm of HU-treated cells [38]. These findings suggest that accumulated Hug1p binds to R2 through interaction with Rnr2p in order to preclude the R1-R2 association in the cytoplasm, leading to the attenuation of RNR activity in post-S-phase or stress conditions. *S. cerevisiae* may concomitantly exploit the allosteric inhibition of RNR and the Hug1p-mediated pathway as feedback regulatory mechanisms for the fine-tuning of intracellular dNTP pools in the absence of Sml1p and Dif1p. RNR activity is tightly regulated because an imbalanced, excessive, or insufficient supply of dNTPs markedly increases the mutation rate during DNA replication and repair, which may cause genomic instability and cell death [19]. A previous study reported that dNTP synthesis was strongly activated in cancer cells, and the development of anticancer drugs targeting RNR is currently in progress [40]. The regulation of RNR activity by three related proteins is a unique and inventive mechanism to maintain genetic fidelity in *S. cerevisiae*. This may provide important information for the discovery of novel drugs.

### DNA DSB repair in *S. cerevisiae*

**Fig. 5:**
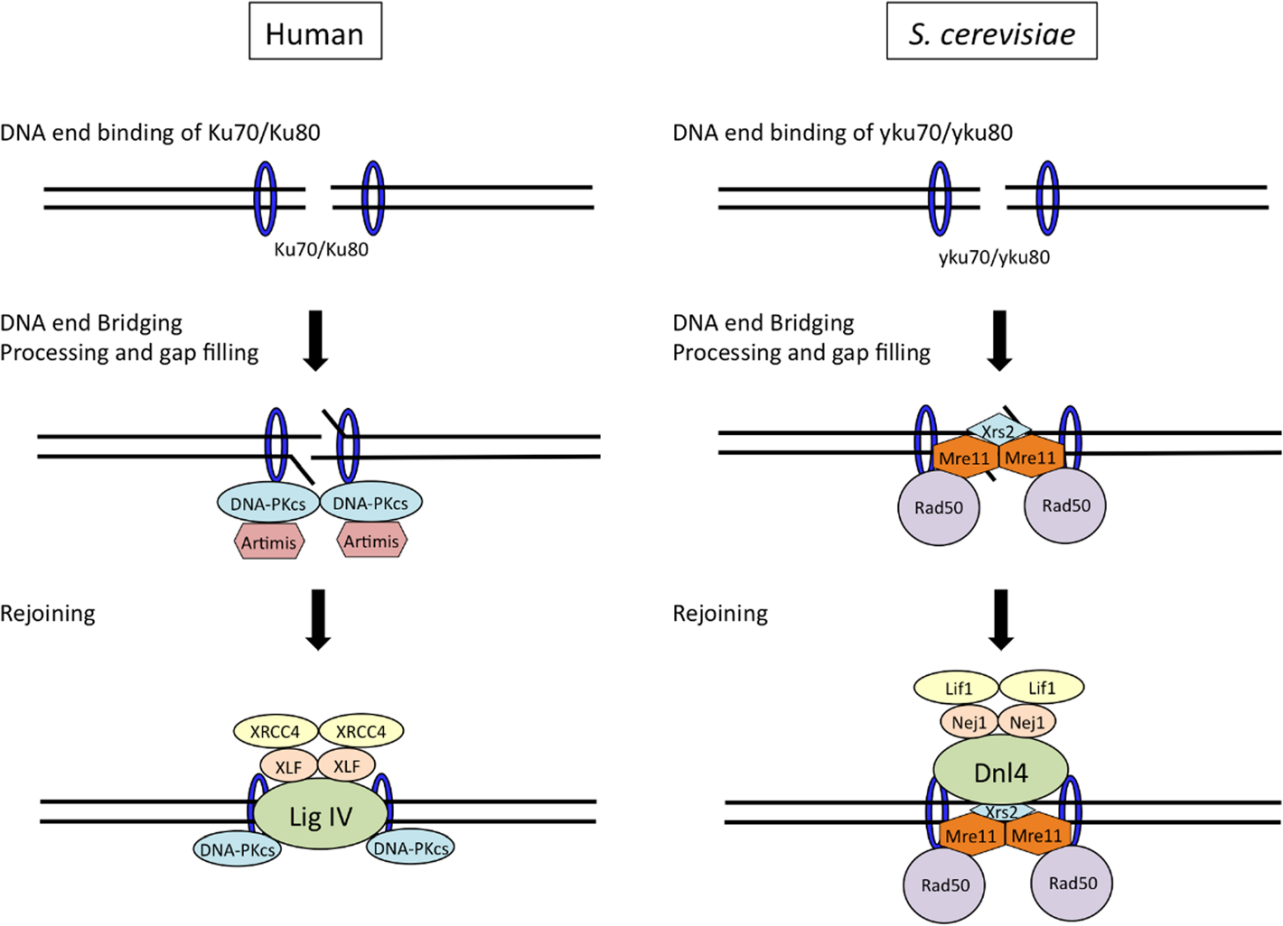
Molecular mechanism of NHEJ in humans and *S. cerevisiae*. When a DSB is created, the NHEJ reaction is initiated by the binding of the Ku protein (the heterodimer of Ku70 and Ku80) to the end of the DSB in both humans and *S. cerevisiae* (top). In humans, Ku subsequently recruits a complex of the DNA-dependent protein kinase catalytic subunit (DNA-PK_cs_) and Artemis, and undergoes end-processing to make ligatable ends (middle). The processed ends are finally joined by a complex containing DNA ligase IV and XRCC4 (bottom). In *S. cerevisiae* that lacks DNA-PK_cs_ homolog, processing of broken DNA ends are carried out by Mre11p-Rad50p-Xrs2p complex (middle), recruiting homologs of DNA ligase IV and XRCC4 for religation (bottom)

In eukaryotes, two main repair pathways have been identified for DNA DSBs which are the most detrimental DNA lesions and can be generated by various stresses such as ROS. Homologous recombination (HR) repairs DSBs by retrieving genetic information from an undamaged homolog, whereas nonhomologous end-joining (NHEJ) pathway rejoins them by direct ligation of the strand ends without any requirement for sequence homology [41]. *S. cerevisiae* mainly uses an HR system of DSB repair, in which exogenous DNA fragments are integrated at homologous sites in the genome if the DNA has a short region of homology at both ends [42]. However, foreign DNA barely integrates into homologous regions on the chromosomes of other organisms. Highly efficient homologous integration is only observed in yeast species of *S.* sensu stricto and *Ashbya gossypii*, a cotton plant pathogen [43]. In order to increase gene targeting efficiencies in other organisms, it is effective to inactivate NHEJ pathway, which is responsible for non-specific integration into the genome (Fig. 5, Table 1). In eukaryotes, it was first reported in the filamentous fungus *Neurospora crassa* that homologous integration efficiency was markedly increased by the inactivation of NHEJ-related genes. *N. crassa* and *S. cerevisiae* have been used in biochemical and classical genetic studies. The genome project of *N. crassa* was completed preceding any other filamentous fungi [44]. By gene disruption of the homologs of Ku70/Ku80 and DNA ligase IV, key components of NHEJ in higher eukaryotes, the frequencies of homologous integration were markedly increased, reaching almost 100% depending on the length of the homologous region on DNA [45, 46]. Thereafter, it became widely known that increases in genomic targeting efficiencies by the inactivation of the NHEJ pathway are common in many filamentous fungi [47–49]. The activity of NHEJ in *S. cerevisiae*, which preferentially utilizes a homologous recombination (HR) system for the genomic integration of foreign DNA, has not yet been elucidated. The radiosensitivity of a homozygous deletion mutant of the *HDF1* gene encoding the Ku70 homolog is similar to that of the diploid wild-type strain. The phenotype of the inactivation of *HDF1* was observed when the *RAD52* gene, encoding a key component for the HR system, was simultaneously disabled. The *rad52*Δ*hfd1*Δ double mutant was more sensitive to ionizing radiation than the *rad52*Δ mutant. In haploid cells, the *hdf1*Δ strain exhibited radiosensitivity during the G_1_- or G_0_-phase, and is considered to be incapable of HR because no homologous chromosome or sister chromatid is available [50]. Previous studies also reported that the expression of Lif1p and Nej1p, homologous to XRCC4 and XLF, respectively, the regulatory subunits of Lig IV, are down-regulated in diploid strains [51–53]. These findings indicate that the contribution of the NHEJ pathway to DSB repair and cell survival after DNA damage is only apparent when HR is unavailable in *S. cerevisiae*. In gene targeting experiments, foreign DNA with homology to target genes, the ideal substrates for HR, are supplied. Under these conditions, *S. cerevisiae* may preferentially operate error-free and secure HR system rather than mutagenic NHEJ. The inactivation of the NHEJ pathway is also expected to increase gene-targeting efficiencies in higher animal cells; however, there is currently no evidence to support this in Chinese hamsters and mice [54–56]. Enhanced gene targeting was subsequently detected in NHEJ-deficient chicken DT40 and human osteosarcoma-derived U2OS cells; however, it was not as efficient as that observed in filamentous fungi [57, 58]. These findings indicated that a microhomology-mediated end joining (MMEJ) pathway, which utilizes very short homology for the repair of DSBs [59], contributes to non-homologous recombination events in animal cells.

**Table 1:** Comparison of NHEJ factors in *S. cerevisiae* and mammalian cells

*S. cerevisiae*	Mammalian
Yku70 (Hdf1)/Yku80 (Hdf2)	Ku70/Ku80
–	DNA-PK_cs_
Dnl4/Lif1	Lig IV/XRCC4
Nej1	XLF
Pol4	DNA polymerase μ or λ
Pso2?	Artemis
Rad27	FEN-1
Mre11/Rad50/Xrs2	Mre11/Rad50/Nbs1

Homologs of most mammalian NHEJ components are also conserved in *S. cerevisiae*; however, this organism lacks DNA-PK_CS_ with DNA-dependent kinase activity, which is recruited to the DNA end by Ku in order to facilitate the rejoining of breaks (Table 1) [60, 61]. In the human U2OS cells described above, the knockdown of DNA-PK_CS_ was more effective at increasing the HR frequency than that of Ku70, Ku80, or Lig IV [58]. It currently remains unclear whether the lack of DNA-PK_CS_ is related to HR selectivity in *S. cerevisiae*, and how DNA-PK_CS_ homologs are conserved through evolution has yet to be clarified.

**Fig. 6:**
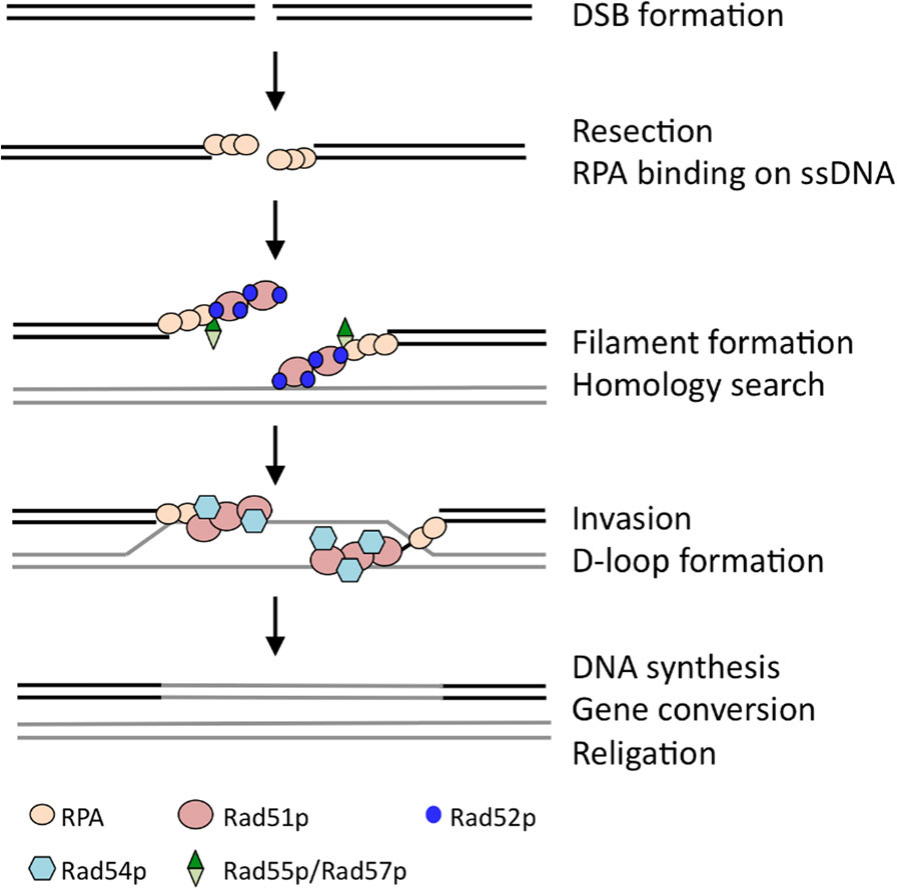
Regulation of HR by factors in Rad52 epistasis group. Upon formation of a DSB, the 3′ single-stranded DNA (ssDNA) end is created by resection. The ssDNA tails are coated by RPA to eliminate secondary structures [62, 63]. Rad52p that possesses multifunctions including the binding activity to both Rad51p and RPA mediates recombination by recruiting Rad51p to RPA-ssDNA complex [64, 65]. Rad52p displaces RPA to enable formation of Rad51p filament extending on the ssDNA, along with Rad55p-Rad57p heterodimer [66–70]. Subsequent genome-wide search for homologous sequences and DNA strand exchange with D-loop formation are accomplished by Rad51p-Rad54p interaction [71–76]. At the end of these processes, Rad54p catalyzes the removal of Rad51p from dsDNA to provide DNA polymerases access for initiation of the repair DNA synthesis reaction

Other aspect that may be correlated to the prominent frequency of HR is unique molecular feature of HR proteins. In *S. cerevisiae*, the process of HR is carried out by the sequential interaction of proteins in Rad52 epistasis group [62–76] (Fig. 6). Rad52p [with ssDNA annealing and binding activities to both replication protein A (RPA) and Rad51p] recruits Rad51p to the 3′ ssDNA coated by RPA, and serves as a seeding site on it by displacing RPA. The Rad51p nucleoprotein filament formation on ssDNA is crucial for the subsequent strand invasion and exchange by interaction with Rad54p. The affinity of RPA for ssDNA is higher than Rad51p, thereby recombination mediator activity of Rad52p is indispensable for Rad51p-dependent HR [77, 78]. The function of these factors are widely conserved through evolution, however, the recombination mediator activity of Rad52p is unique in *S. cerevisiae*. BRCA2 (BReast CAncer susceptibility gene 2) that has little homology with *S. cerevisiae* Rad52p shares the mediator function instead of Rad52 orthologue in human [79, 80]. Consistent with this, chicken and human cell lines lacking Rad52 show no increase in sensitivity to DNA-damaging agents and the efficiency of gene targeting is only marginally reduced [81, 82]. In contrast, *S. cerevisiae rad52* mutants exhibit severe defects in all forms of HR, and recruitment of Rad51p to DSBs is strongly dependent on Rad52p [83]. These findings imply that the unique mediator function of Rad52p may also be relevant to efficient HR in *S. cerevisiae.*

### Unequal mutation frequency in the genome of *S. cerevisiae*

In some occasions, living organisms accept genetic alterations that escaped protection mechanisms for DNA. This may lead extensive phenotypic diversity or variation in clonal populations of microorganisms, and play a role in adaptation to novel environments. The phenotypic variation or instability, which occurs via multiple mechanisms, may be a form of cellular differentiation and a stochastic means for modulating gene expression. We previously reported a case of phenotypic variation in a clinically-derived *S. cerevisiae* strain [84].

**Fig. 7:**
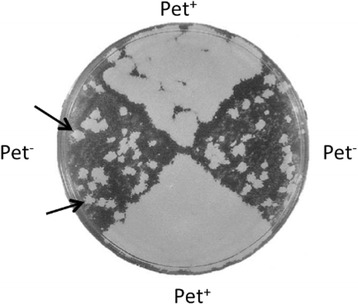
Phenotypic instability of a clinically-derived strain of *S. cerevisiae*. The arrows indicate Pet^+^ colonies appearing from the Pet^−^ progeny

**Fig. 8:**
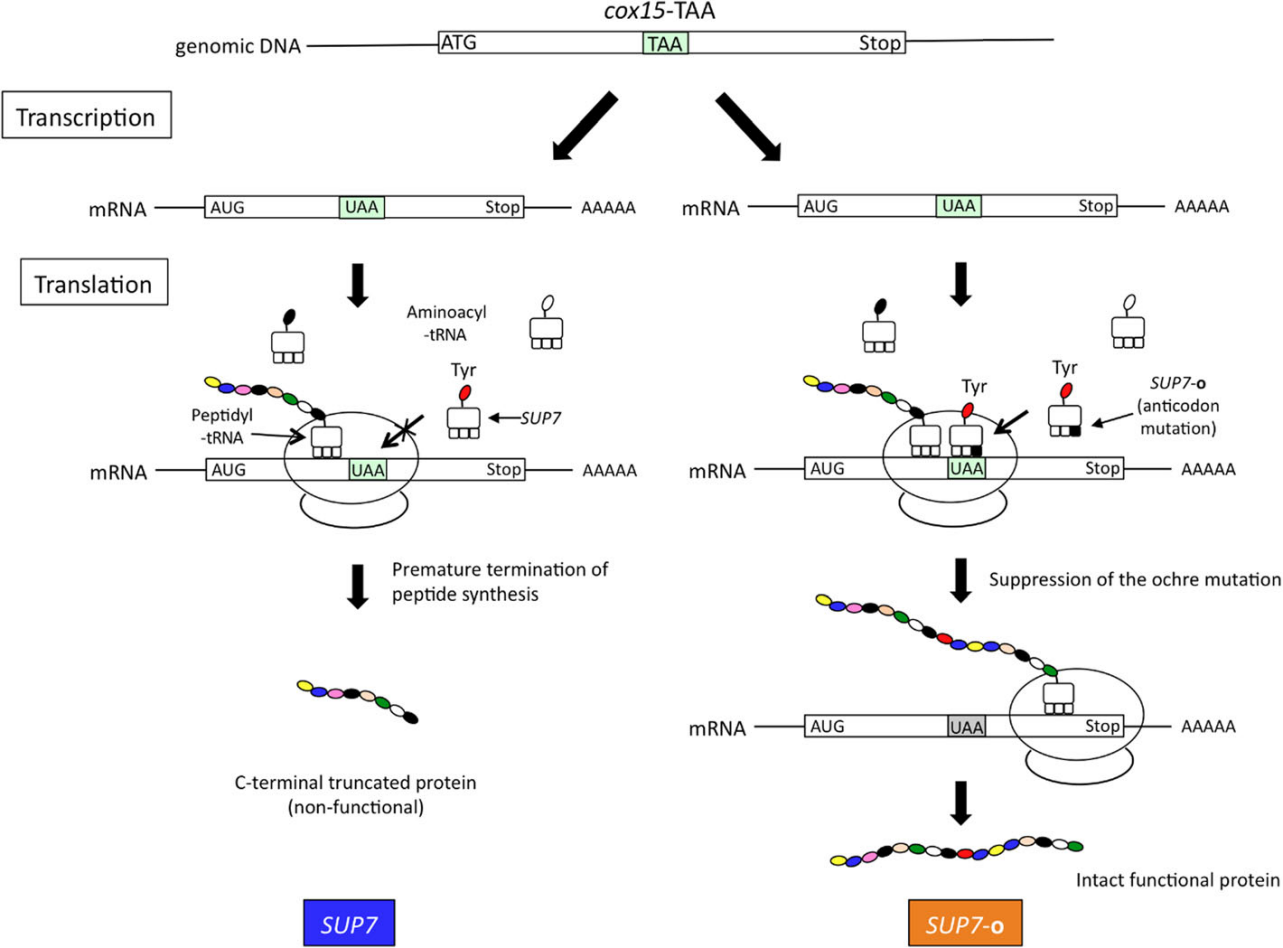
Molecular mechanism of the suppression of the *cox15-*TAA mutation by *SUP7-* **o**. In a cell containing wild-type *SUP7*, ribosome ceases translation elongation when it reaches the in-frame ochre mutation to produce a C-terminal truncated non-functional polypeptide (left). When an anticodon mutation occurs in the *SUP7* gene, the ochre suppressor *SUP7-* **o** inserts tyrosine to suppress the ochre codon, and enables translation elongation to proceed in order to produce a full-length polypeptide (right)

**Fig. 9:**
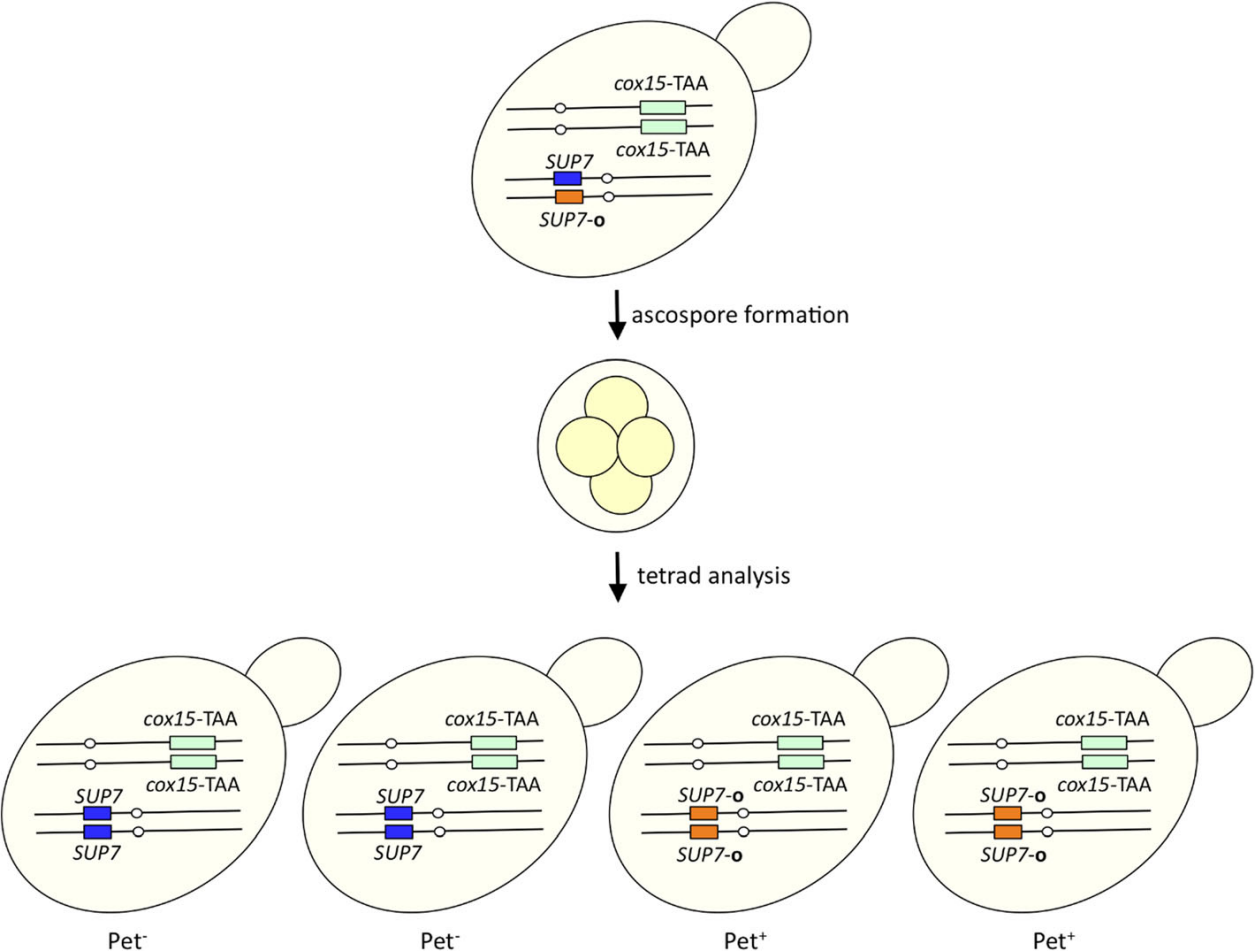
Segregation pattern of mutations and the Pet phenotype in progenies of YJM421 YJM421 was homozygous for *cox15*-TAA and heterozygous for *SUP7-* **o**. The Pet^+^ phenotype and *SUP7-* **o** were co-segregated in progenies [84]. YJM421 is a homothallic strain in which haploid ascospores undergo mating-type switching to become diploid during propagation after germination

In addition to industrial or laboratory strains, *S. cerevisiae* has been isolated as an opportunistic pathogen from immunodeficient patients [85, 86]. The dissected tetrad of the clinically derived diploid strain YJM421 shows Mendelian (2:2) segregation for its ability to respire on agar plates containing non-fermentable carbon sources such as glycerol and ethanol. The respiration deficiency phenotype (Pet^−^) is unstable and Pet^−^ segregants frequently produced Pet^+^ colonies after prolonged incubations (Fig. 7). Genetic and molecular genetic analyses of YJM421 revealed that the Pet^−^ phenotype is due to the ochre mutation [CAA (glutamine) to TAA at codon 39] in *COX15* ORF (*cox15-*TAA), which encodes a protein required for cytochrome C oxidase assembly in mitochondria [87]. In Pet^+^ progenies, the ochre suppressor mutation was found in *SUP7* (*SUP7-* **o**), one of the tRNA-Tyr genes to suppress *cox15-*TAA (Fig. 8). These observations confirmed that YJM421 is homo- and heterozygous for *cox15-*TAA and *SUP7-* **o**, respectively, and the segregation of Pet^+^ progenies is consistent with the segregation pattern of *SUP7-* **o** (Fig. 9).

**Fig. 10:**
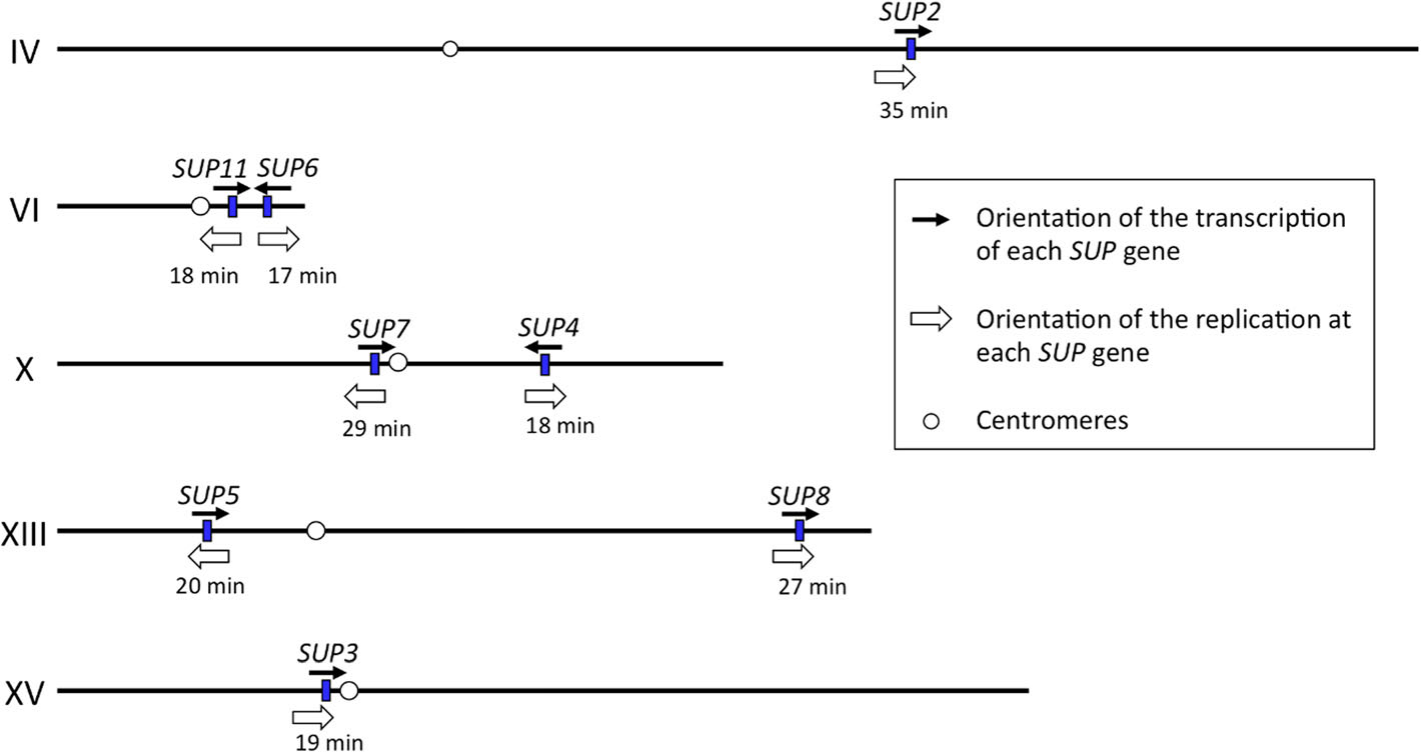
Chromosomal localization of tRNA-Tyr genes. Eight tRNA-Tyr genes are dispersed in the genome. The orientation of transcription of the each *SUP* gene is shown by black arrow. Direction of DNA replication is represented by open arrow with timing (minutes) during S-phase based on data reported by Raghuraman et al. [112]

**Fig. 11:**
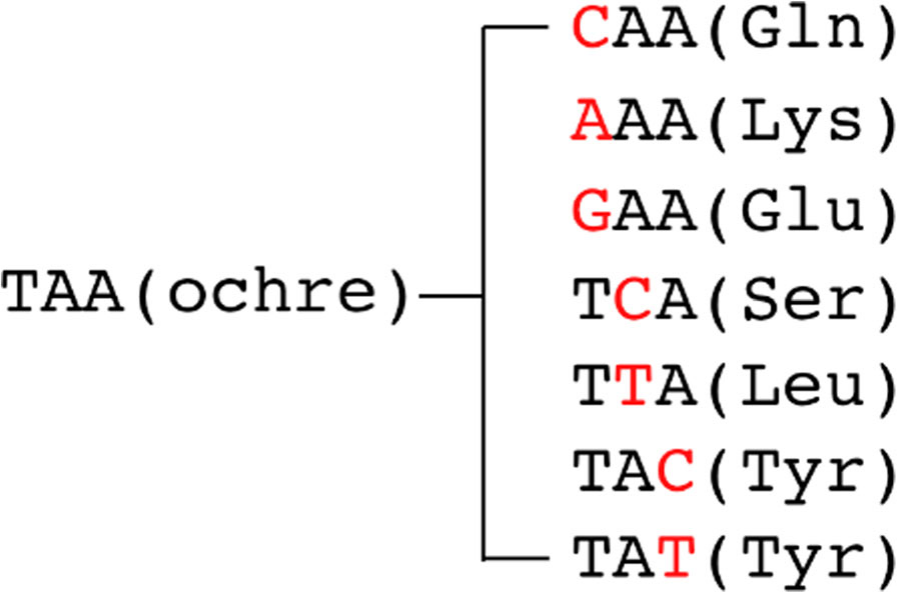
Patterns of reversions from the *cox15-*TAA mutation. The nucleotide written in red is the mutated position with coding amino acids in parentheses. Cox15 proteins carrying these reversions are functional [84]

*S. cerevisiae* possesses eight tRNA-Tyr genes that are dispersed on the genome (Fig. 10). We developed an easy and rapid genotyping method to identify the ochre suppressor mutation within the eight tRNA-Tyr loci, and examined the frequencies of the tyrosine-inserting ochre suppressor mutation (*SUP-* **o**) and reversion of *cox15-*TAA. More than 40 spontaneous Pet^+^ mutants were isolated from each of the three parental Pet^−^ strains, diploid and haploid strains derived from YJM421 and a haploid laboratory strain, and mutants were genotyped to identify the tRNA-Tyr locus mutated to *SUP-* **o**. In cases in which all tRNA-Tyr loci were the wild-type, *cox15*-TAA was sequenced. In all 129 Pet^+^ mutants, *cox15-*TAA reversion was only observed in three mutants, while the *SUP-* **o** mutation at one of the eight tRNA-Tyr loci was responsible for conferring the Pet^+^ phenotype in others. There were seven patterns of single base-pair substitution mutations that convert a TAA ochre codon to one of the seven sense codons, and all of them resulted in a functional Cox15p (Fig. 11) [84]. Furthermore, although *SUP-* **o** mutations at other tRNA loci may suppress *cox15-*TAA, these mutations occurred almost exclusively at tRNA-Tyr loci in spontaneous Pet^+^ mutants. The distribution of 42 *SUP-* **o** mutants within tRNA-Tyr loci isolated from each of the three parental Pet^−^ strains was summarized in Table 2. Aside from a single base-pair polymorphism within the intron, all members of the tRNA-Tyr gene family had identical sequences. We observed biased mutation frequencies among them that were highly significant in the χ^2^ test (*p* < 0.001). The mutation frequency at the *SUP6* locus (approximately 30%) was markedly higher than the average frequency, while those at *SUP2* /*SUP8* and *SUP3* loci were markedly lower (1.6% and 5.6%, respectively). Our study on *S. cerevisiae* revealed mutation rate variations at different loci, suggesting that the mutation rate is not uniform across the genome.

**Table 2:** Number of independently isolated *SUP-* **o** mutants

Strain	*SUP2-* **o**	*SUP3-* **o**	*SUP4-* **o**	*SUP5-* **o**	*SUP6-* **o**	*SUP7-* **o**	*SUP8-* **o**	*SUP11-* **o**
YSA3	1	4	7	4	11	7	1	7
V1–1-	1	2	3	8	11	7	1	9
S183	0	1	10	5	17	5	0	4
Total	2	7	20	17	39	19	2	20

YSA3 and V1–1- are diploid and haploid, respectively, derived from the YJM421 strain, and S183 is the haploid strain in the laboratory background (data referred from [84])

All three parental strains showed similar results with respect to mutation rate variations within tRNA-Tyr genes, which suggest that this is a common phenomenon in *S. cerevisiae* rather than being background-dependent (clinically-derived strain vs. laboratory strain). In order to deduce the mechanism underlying the position effects on mutation frequencies, we examined the relationship between tRNA-Tyr locus-specific mutation frequencies and multiple factors. We examined the suppression efficiencies of *SUP-* **o** mutants using a reporter plasmid that is a measurable readthrough of the ocher mutation. We also searched previous studies; the distances of tRNA-Tyr genes from the centromere or telomere, the localization of transposons, the expression levels of flanking genes, the timing of replication, or the rate of replication fork movement, and found that none correlated with the biased mutation frequency. However, it is interesting to note that *SUP2*, *SUP3*, and *SUP8*, the three loci with the lowest mutation frequencies, were all transcribed in the same direction as replication forks. In contrast, *SUP4*, *SUP5*, *SUP7*, and *SUP11*, the four loci with average mutation frequencies, as well as *SUP6*, the locus with the highest mutation frequency, were all transcribed in the opposite direction to replication forks (Fig. 10). This finding suggests that gene orientation relative to the direction of replication may be a factor explaining locus-specific mutation frequencies. However, one or more additional factors may contribute to biased mutation frequencies [84].

**Fig. 12:**
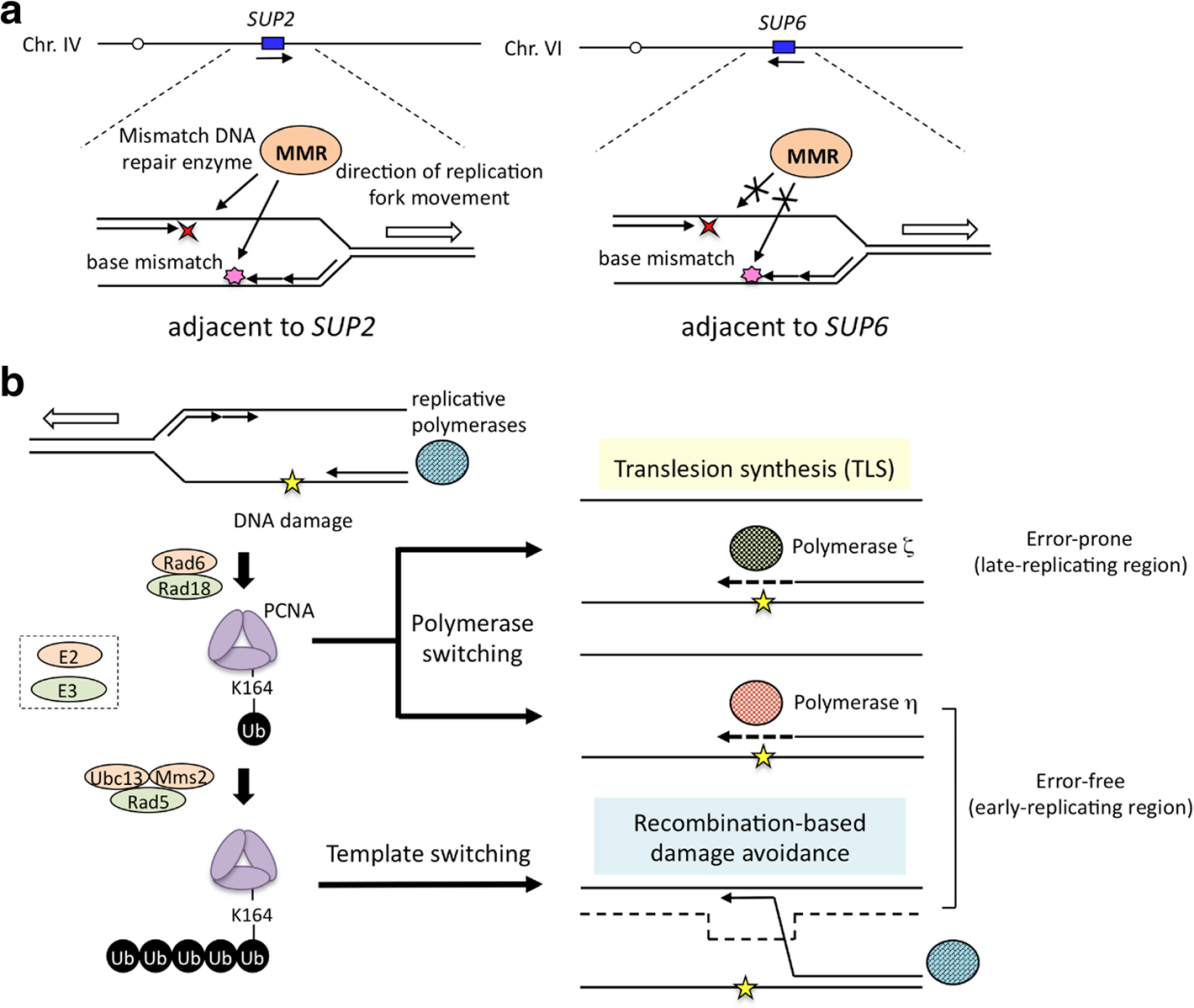
Putative mechanisms that contribute to the bias of the mutation rate in the genome. **a** Difference in the efficiency of the mismatch DNA repair. Mismatch base-pairing by damage-induced replication errors is rapidly repaired in the region adjacent to *SUP2*, whereas efficiency is lower in the *SUP6* adjacent region [88]. **b** Involvement of replication timing on the chromosome. Fidelity of DNA damage bypass may be determined by availability of TLS polymerases (error-free or error-prone) and induction of Rad5p-mediated recombination-based template switching during cell cycle, those are correlated with ubiquitination status of PCNA (see text for detail) [89, 90, 92–96]

The unequal mutation frequency/rate across the *S. cerevisiae* genome was verified in studies by other groups [88, 89]. In order to detect frameshift mutation rates, Hawk et al. [88] constructed 10 isogenic yeast strains carrying the fusion reporter gene, the uracil synthetic gene *URA3*, including in-frame microsatellite GT-repeats (*URA3*-*GT*) at different locations in the genome. They examined insertions at the *SUP2* and *SUP6* loci showing the lowest and highest mutation frequencies, respectively, in order to convert to the ochre suppressor [84]. The rate of frameshift mutations was 16-fold different among these strains, and this rate was the highest at the *SUP6* locus, which was 9-fold higher than that at the *SUP2* locus. These findings suggest that frameshift mutations as well as base substitution mutations preferably occur at the *SUP6* locus. In mismatch-repair (MMR)-deficient strains that lack Msh2p, the rates of frameshift mutations were elevated at all loci tested; however, differences among the mutation rates at different genomic sites were markedly less than those observed in the MMR^+^ wild-type strains (from 16-fold to 2-fold). These findings indicate that a difference in MMR efficiency is one of the factors affecting the mutation rates at different loci in the genome (Fig. 12a) [88].

Lang et al. [89] examined the relationship between chromosomal localizations and spontaneous mutation rates by measuring the mutation rate of the *URA3* gene integrated at 43 different locations tiled across chromosome VI in *S. cerevisiae*. Two tRNA-Tyr genes, *SUP6* and *SUP11* were localized on chromosome VI. They showed that the mutation rate varied 6-fold across a single chromosome. Variations in the mutation rate correlated with replication timing, with earlier-replicating regions having lower mutation rates and regions replicated in the late S-phase having higher mutation rates. The manipulation of replication timing by deleting the earliest and most efficient origin *ARS607* increased the mutation rate at the *URA3* reporter gene by 30%. Furthermore, the disruption of gene encoding translesion synthesis (TLS) DNA polymerase Rev1p resulted in a 4.8-fold reduction in the mutation rate at the late-replicating locus with the high mutation rate [89]. TLS polymerases have the ability to synthesize DNA strand past lesions with markedly higher error rates than replicative polymerases [90, 91]. DNA damage-induced replication fork stall triggers monoubiquitination of proliferating cell nuclear antigen (PCNA encoded by *POL30* gene) at K164, which is catalyzed by the E2 ubiquitin (Ub) conjugating enzyme Rad6p and the E3 Ub ligase Rad18p (Rad6p/Rad18p complex) [92]. Monoubiquitinated PCNA is known to activate TLS polymerases Rev1p and Rad30p (polymerase η) [93, 94]. Rev1p assists to recruit other TLS polymerases Rev3p and Rev7p consisting of error-prone polymerase ζ, in addition to its own TLS polymerase activity [90]. Rev1p is not expressed until the late S-phase, while error-free polymerase η is constantly expressed [95]. Mutation rate bias associated with replication timing may be determined by the availability of TLS polymerases during cell cycle. DNA replication fork stall also induces recombination-based template switching, another postreplication repair pathway, involved in polyubiquitinated PCNA at the same K164 residue catalyzed by Ubc13p-Mms2p-Rad5p Ub E2-E3 complex [92]. Rad5p also has DNA helicase activity specific for replication fork regression, which is thought to facilitate sister strand recombination [96]. These findings suggest that DNA damage generated in early-replicating regions may be bypassed by recombination-based template switching or polymerase η-involved error-free TLS by polymerase switching, while damaged bases in late-replicating regions are more likely to be subjected to mutagenic TLS by polymerase ζ, (Fig. 12b) [89].

These findings are based on the measurement of spontaneous mutation rates. It is of interest to examine how mutation rates change when induced by ionizing radiation and various DNA-damaging agents.

### Nonsense suppressors and phenotypic variations: Nonsense suppressors are agents for environmental adaptation?

The mutations that convert tRNA-Tyr genes to ochre suppressors described above confer phenotypic variations to Pet^+^ cells. The tRNA-Tyr *SUP-* **o** and amber suppressor *SUP-* **a** affect cell proliferation, ascospore formation, and sensitivity to high osmolality. Variations in phenotypes correlated with the suppression efficiency of each *SUP-* **o**/*SUP-* **a** mutant [84, 97–99]. Therefore, nonsense suppressors may act on nonsense mutations such as the termination codons of other genes and natural nonsense mutations that spontaneously arose within protein coding regions during the evolutionary process, as well as nonsense mutation reporters (in the case of our study, a *cox15-*TAA mutation). The translational readthrough of termination codons by nonsense suppressors extended the C terminus of nascent polypeptides, which may modulate the activities of cellular proteins [100]. The suppression of natural nonsense mutations may lead to the production of full-length functional proteins from nonsense-containing transcripts (Fig. 8) [84]. Nonsense suppressors may alter global gene expression profiles, such as DNA repair, DNA damage response, and metabolism, in addition to ascospore formation and high osmolality tolerance, as described above.

**Fig. 13:**
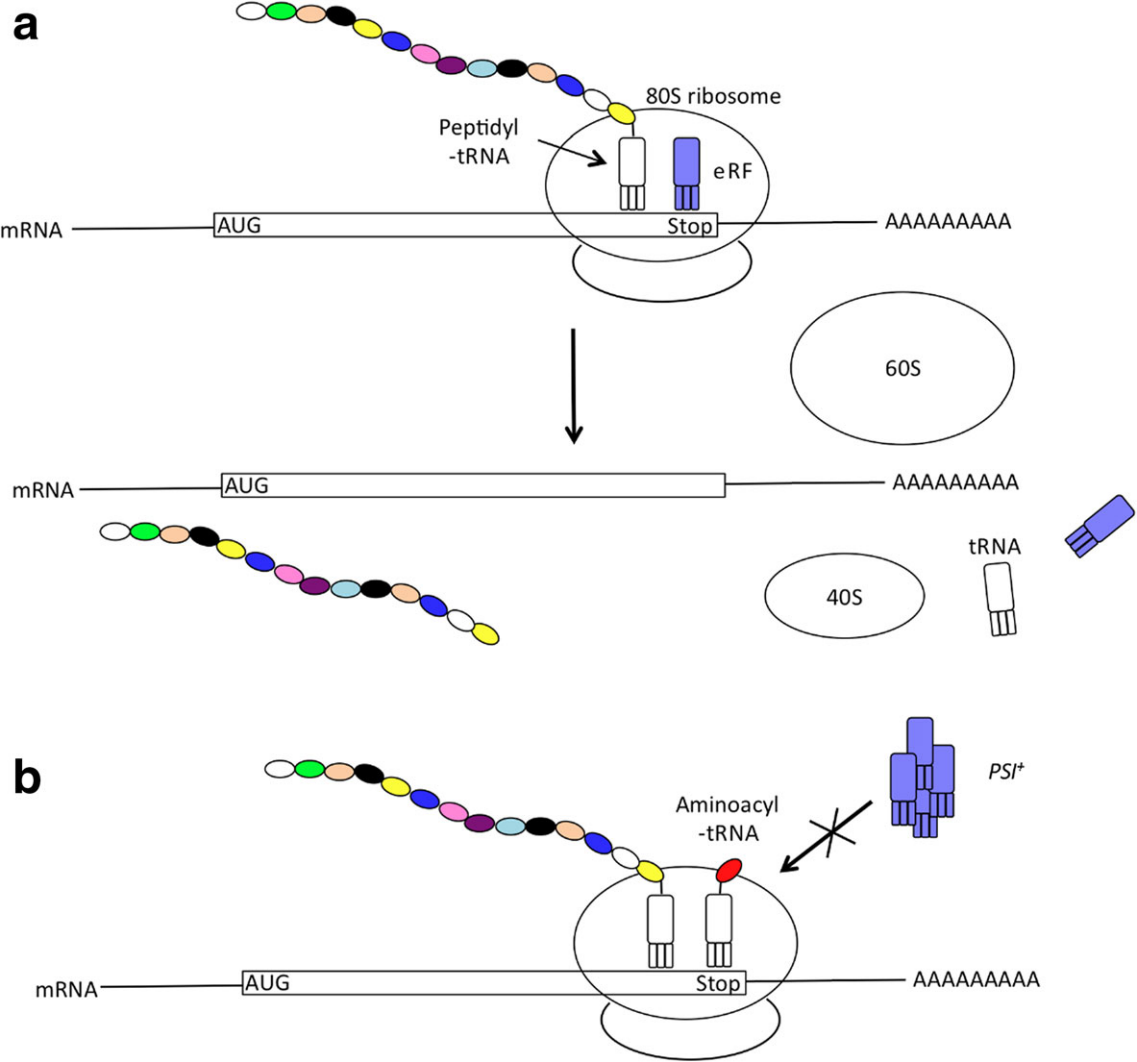
Decrease in translation termination efficiency by the ψ (*PSI)* factor. **a** Translation termination in normal cells. When actively translating 80S ribosomes reach the termination codon on mRNA, eRF instead of aminoacyl-tRNA enters into the A site of ribosomes. Translation then ceases, leading to the dissociation of the nascent polypeptide, tRNA, and eRF from ribosomes. Ribosomes themselves also dissociate to 40S and 60S subunits. **b** *PSI* ^+^ generation by the aggregation of Sup35. Since functional Sup35 is depleted, the efficiency of translation termination is decreased, allowing the entry of aminoacyl-tRNA into the ribosome A site. Ribosomes continue to translate the 3′-UTR of mRNA beyond the intrinsic termination codon. This may cause the C-terminal extension of nascent polypeptides, as well as the suppression of natural nonsense mutations

The readthrough of nonsense codons also occurs by mutations in the *SUP35* and *SUP45* genes, which encode the translational termination factors (eukaryotic release factor: eRF) eRF3 and eRF1, respectively. Sup35p and Sup45p interact to form functional eRF [101]. The factor ψ (*PSI*), which was identified as a modifier of nonsense suppression in some strains of *S. cerevisiae*, is a non-Mendelian (cytoplasmic) element [102]. A subsequent study revealed that Sup35p itself forms amyloid-like fiber-shaped aggregates when overproduced. This form of Sup35p is referred as *PSI* ^+^, a prion form in *S. cerevisiae* [103]. In these mutants, the recognition of a termination codon is impaired by the depletion of functional eRF, leading to continuous translation beyond termination codons (Figs. 8 and 13). While the suppression efficiencies of nonsense codons by aberrant eRFs are lower than the nonsense suppressor form of tRNA-Tyr, aberrant eRFs are omnipotent suppressors that act on all types of nonsense codons (ochre, amber, and opal) [104, 105]. The conversion of Sup35p to *PSI* ^*+*^ gave rise to phenotypic variations associated with translation termination efficiency upon adaptation to environmental conditions [106–110].

The numbers of each termination codon assigned for all 6221 ORFs are 2948 for ochre (TAA) (47.4%), 1416 for amber (TAG) (22.8%), and 1857 for opal (TGA) (29.8%) [111]. The numbers of genes affected by nonsense readthrough will vary depending on the type and efficiency of the nonsense suppressor, which may confer diverse phenotypes. The acquisition of phenotypic variations by nonsense suppressors may be advantageous for adaptation to environmental stresses and a strategy to maintain the species. It may also be a powerful driving force for evolution.

## Conclusions

The generation of DNA lesions impairing accuracy of genetic information are surveyed and repaired by evolutionarily conserved mechanisms in eukaryotes, with somewhat divergent machineries and/or activities specific to *S. cerevisiae*. The evolution of molecular architecture may be elucidated by cross-species comparison among related organisms. Meanwhile, genetic alteration that can confer substantial phenotypic variation plays significant roles for adaptation against hazardous environmental stresses. Yeast genetics will be a powerful tool to unveil the molecular mechanisms underlying each case of environmental adaptation.

In *S. cerevisiae,* many strains were isolated from different origins such as brewery, laboratory, and clinical origins, and a genome project was accomplished for each of these strains [111]. Similarly, genome projects for yeast species in the group of *Saccharomyces*, or “budding yeast” in a broad sense, such as dimorphic fungi *Candida albicans* and *Cryptococcus neoformans* that grow by budding in a part of the life cycle, have also been performed. Abundant resources are now available, and comparative genomics have contributed to advances in yeast research. By taking advantage of these circumstances, post-genome research to approach as yet unrevealed mysteries of life may be facilitated in yeast. We expect exciting research on the unique features of *S. cerevisiae* to advance in the future.

## Abbreviations

dNTP: Deoxyribonucleoside triphosphate
DSB: Double-strand break
eRF: Eukaryotic release factor
HR: Homologous recombination
HU: Hydroxyurea
IR: Ionizing radiation
LET: Linear energy transfer
MMEJ: Microhomology-mediated end joining
MMR: Mismatch repair
MMS: Methyl methanesulfonate
NHEJ: Non-homologous end joining
PCNA: Proliferating cell nuclear antigen
RNAi: RNA interference
RNR: Ribonucleotide reductase
ROS: Reactive oxygen species
RPA: Replication protein A
TLS: Translesion synthesis
Ub: Ubiquitin
UV: Ultraviolet
